# Isolation and characterization of cyanobacteria and microalgae from a sulfuric pond: Plant growth-promoting and soil bioconsolidation activities

**DOI:** 10.3934/microbiol.2024041

**Published:** 2024-11-08

**Authors:** Beatrice Farda, Rihab Djebaili, Enrico Sabbi, Giancarlo Pagnani, Paola Cacchio, Marika Pellegrini

**Affiliations:** 1 Department of Life, Health and Environmental Sciences, University of L'Aquila, 67100 L'Aquila, Italy; 2 Department of Bioscience and Technologies for Food, Agriculture and Environment, University of Teramo, Teramo, 64100, Italy

**Keywords:** cyanobacteria, microalgae, sulphuric pond, metabarcoding, plant growth-promoting, sunflower, salinity, bioconsolidation, biostimulant

## Abstract

Sustainable alternatives are essential to improving agriculture production to meet the growing world's critical demands. Cyanobacteria and microalgae are considered renewable resources with a wide range of potential uses in the agricultural sector. We aimed to isolate cyanobacteria and microalgae from the mud of a carbon dioxide-rich sulfur pond and to investigate their plant growth-promoting (PGP) and soil bio-consolidating ability. Mud samples were subjected to DNA extraction and 16S rRNA gene sequencing to characterize the prokaryotic community. Phototrophic culturable microbiota was isolated and evaluated for different PGP properties. The most relevant isolates were combined in a consortium and used for *in vitro* bioconsolidation activity. In a greenhouse experiment, the isolates were evaluated for their ability to promote salinity stress tolerance in sunflower plants. Metabarcoding results showed that most Amplicon Sequence Variants (ASV) were associated with Actinobacteriota (35%), Proteobacteria (19%), and Acidobacteriota (11%) at the phylum level and *Unknown* (32%) and uncultured (31%) lineages at the genus level. The culture-dependent method yielded eight isolates associated with cyanobacteria and microalgae genera. The isolates obtained showed interesting PGP activities. Isolates C1, C2, and M1 were selected based on phosphate solubilization (85.6 µg PO_4_^3−^ mL^−1^ on average), indoles (C1 and M1 0.54 µg mL^−1^ IAA equivalents on average), and ACC deaminase activity (C2 and M1 6.00 µmol α-KB mg proteins^−1^ h^−1)^. The consortium efficiently consolidated sand particles in the presence of calcium carbonate by forming biomineralized aggregates. *In planta* results showed positive effects of the consortium on *Helianthus annuus L*., plant growth under normal conditions and salt stress. The positive effects on soil and plants indicated their effectiveness as bioconsolidants and biostimulant agents. Our findings highlight the interesting potential of cyanobacteria and microalgae applications in sustainable agriculture.

## 1. Introduction

Global climate change, population growth, plant diseases, and poor soil significantly impact agricultural production [1]. Crop productivity has dramatically increased over time due to the invention of chemical products such as synthetic fertilizers and insecticides. Unfortunately, the widespread use of such chemical products harms the ecosystem and the environment [1],[2]. Modern agriculture faces a serious challenge: Increasing agricultural production while preserving the quality of the environment and using the resources available to meet the population's food needs [3]. The “Green Revolution” approach also increases agricultural and reduces chemical-based fertilizers' environmental and human health risks. The current approach to sustainable agriculture emphasizes environmentally friendly, low-cost farming that uses local microorganisms to strengthen the agroecosystem's resilience, self-regulation, and ability to maintain productivity and profitability [4],[5]. Recent research suggests that cyanobacteria and microalgae can be used as bio-agents to improve agricultural productivity, reduce greenhouse gas emissions, and restore the ecological integrity of degraded soils [3],[6],[7].

Cyanobacteria (prokaryotic) and microalgae (eukaryotic) are unicellular microscopic oxygenic phototrophs that are among the most abundant organisms on Earth in terms of quantity and are found in most illuminated habitats [4],[8]–[10]. Cyanobacteria have been inaccurately called blue-green algae because of their color. Phylogenetically, they are related to the bacteria domain [11]. They are often small and grow in massive colonies. Cyanobacteria comprise a wide range of bacteria of different sizes and shapes. They are found in 150 of the currently recognized genera [8]. Cyanobacteria are a class of ancient microbes that first appeared between 2.6 and 3.5 billion years ago [8],[12]. Microalgae can grow and develop rapidly by effectively absorbing carbon dioxide (CO_2_) and nutrients such as phosphorus and nitrogen from the water [13]. Cyanobacteria and microalgae have many uses and applications and are responsible for a significant proportion of the world's CO_2_ and N_2_ fixation [10],[14]. They serve as model organisms for easy genetic manipulation [15],[16]. These microorganisms are cost-effective bioresources that have been used for a variety of industrial applications as food supplies, photoprotective compounds, bioplastics, dyes, and colorants, and they can produce biofuels and act as a food source, environmental bioindicators and bioremediation, and agriculture bio fertilizers [17]–[20]. They can also synthesize compounds of pharmaceutical interest (antimicrobial, antiviral, anticancer, antiprotozoal, etc.) [10],[21].

Although cyanobacteria and microalgae can exist independently in nature, some are found in symbiosis with various eukaryotic hosts, such as plants, fungi, sponges, and protists [22]–[24]. Cyanobacteria and microalgae play an important role in the biogeochemical cycles of oxygen, nitrogen, and carbon, a crucial process in agricultural systems [7]. In the case of non-photosynthetic hosts, cyanobacteria, which are often filamentous, fix N_2_ in specialized cells called heterocysts [17],[25]. By fixing N_2_, cyanobacteria, and microalgae meet their nitrogen requirements and produce specific bioactive molecules that improve soil nutrient status, plant growth, and protection against disease [3],[7]. As a fertilizer, cyanobacteria and microalgae can also release plant growth hormones as secondary metabolites and assist in transporting nutrients from the soil to plants, promoting soil aggregation and chemical properties [13],[26]. Phosphorus is the second most important nutrient for plants and soil microbes after nitrogen. In this context, it has also been reported that cyanobacteria and microalgae can solubilize and mobilize the insoluble organic phosphates present in the soil using phosphatase enzymes, thereby improving the bioavailability of phosphorus for plants [10]. However, thanks to the production of plant hormones such as auxins, cytokinins, and gibberellins, cyanobacteria and microalgae are not only able to increase the acquisition of nutrients by plants, but they also actively promote their germination, growth, and development [26]–[29]. In addition, cyanobacteria and microalgae can produce bioactive compounds, including sulfated exopolysaccharides (EPS), which benefit soil structure and plant growth [1],[30]. Extracts of microalgae and cyanobacteria have been reported to enhance plant development and nutrient uptake, thereby increasing crop growth and yield [31],[32]. Dry biomass of various cyanobacteria, also known as “bio-inoculation” or “algalization”, has been applied to the soil since the 1950s to improve plant development, health, and yield [33],[34].

Building on unstable ground is a natural consequence of the global shortage of available land. Low strength and high compressibility are two common characteristics of these soils [35]. Traditionally, engineered fill has been used to replace low-strength soils. Chemical grouting is currently gaining popularity as a cost-effective alternative. Several additives produce chemical grouts, such as Portland cement, lime, asphalt, sodium silicate, acrylate, lignin, urethane, and resins [36]. Although many of these additives are effective, they often alter the soil's pH and potentially contaminate soil and groundwater [35],[37]. In the soil, cyanobacteria and microalgae act in the upper crust, releasing exopolysaccharides that bind soil particles together. This leads to soil aggregation, the accumulation of organic matter, and an increase in the water-holding capacity of the upper soil layer. As a result, cyanobacterial and microalgal growth improves soils' physical and chemical properties, and the produced exopolysaccharides can aid in the recovery and improvement of infertile soils [7],[33],[38]. Cyanobacteria and microalgae produce “biocrusts”, which are agglomerations of soil particles made of microorganisms, lichens, and bryophytes [4],[39]. Biocrusts are a common element in drylands, occupying 40 to 100 percent of the interplant gaps and contributing to important ecosystem services [40]. Because of their recognized roles, biocrusts have been identified as relevant communities for effectively restoring disturbed drylands [41]. Among biocrust communities, cyanobacteria and microalgae are pioneer species that improve soil quality and allow other organisms to colonize the area [39],[42]. As a glue, cyanobacterial filaments and their extracellular secretions, consisting mainly of exopolysaccharides (EPS), bind soil particles and promote the formation of soil aggregates, thus increasing soil stability [4]. Soil texture influences biocrust formation, structure, and water dynamics. It has been reported that most cyanobacterial inoculation trials have been conducted on sandy soils [43],[44].

It is known that environments that have not been previously explored may contain microorganisms with important characteristics. In this study, we hypothesized the role of microorganisms in CO_2_-rich sources. For this purpose, the sludge from the CO_2_-rich sulfur pond was processed for DNA extraction and 16S rRNA gene sequencing to characterize the prokaryotic community. The sludge samples were used to isolate and characterize cyanobacteria and microalgae for different PGP activities and soil bioconsolidation capacity. The consortium of the most relevant isolates (C2, C3, and M1) was used for the *in vitro* bioconsolidation activity and *in planta* experiment on *Helianthus annuus* to assess the inoculation effects on plant growth and development parameters.

## 2. Materials and methods

### 2.1. Soil sampling

The sulfuric pond called “La Fermentina” in the San Donato Val di Comino municipality (41°41′01.89″N, 13°47′19.55″E) served as the sampling site for this study. Five subsamples containing both liquid and sludge were taken from the formed photosynthetic biofilms and placed in sterile containers. The samples were kept in a cool sampling bag and transported to the laboratory. Subsamples were thoroughly mixed to obtain a composite sample. The culturable and molecular approaches were carried out on three aliquots of the composite sample, each processed independently.

### 2.2. DNA extraction and 16S rRNA gene metabarcoding

The DNA was extracted using the NucleoSpin® Soil kit (Macherey Nagel, Germany) according to the manufacturer's instructions. A Nanodrop spectrophotometer (Thermo ScientificTM, Waltham, MA, USA) and a Qubit fluorometer (Thermo ScientificTM, Waltham, MA, USA) were used to determine DNA's amount and purity. The independent replicates for each sample were mixed in an equimolar ratio. MiSeq Illumina technology (Bio-Fab Investigation, Rome, Italy) performed a paired-end 16S rRNA community sequencing focusing on the V3 and V4 regions of the 16S rRNA gene [45],[46]. After filtering, the reads were counted and checked for reliability. The DADA2 plugin was used to generate ASV (Amplicon Sequence Variant) using QIIME2 (qiime2-2020.2 version) [47]. The 16S file from the SILVA database (https://www.arb-silva.de/ accessed on 14 October 2021) was used to select the V3-V4-specific region and applied to instruct the classifier using the fit-classifier-naive-Bayes plugin.

### 2.3. Isolation and characterization of culturable phototrophic microbiota

Phototrophic microbiota was isolated on a solid BG11 culture medium using several dilutions up to 10^−4^ [9],[48]. In addition, enrichment cultures were prepared from all samples in liquid BG11. Incubation was performed in a climatic growth chamber (28 °C, 12 h photoperiod, lightening 150–200 µmol (photon) m^−2^ s^−1^). Enrichment cultures and biomass aggregates developed on the solid medium were subcultured several times until individual colonies appeared pure. Microscopic examination of isolates allowed a preliminary identification based on the morphotype. Based on cells dimension, shape and reactivity to Lugol staining and fluorescent microscope observations, isolates were associated with cyanobacteria and microalgae genera. Each qualified isolate was given a unique identification number (ID). A total of eight isolates were collected. The isolates were grown in flasks and small bioreactors using a BG11 liquid medium. After uniformity and purity controls, the isolates were stored on BG11 agar slants and glycerolates (50% v/v, −80 °C storage) in the Environmental Microbiology Culture Collection (LMUNIVAQ) [9].

### 2.4. Plant growth-promoting activities

#### 2.4.1. Phosphate solubilization

To assess the ability to solubilize phosphate, the various isolates were inoculated on liquid NBRIP medium (National Botanical Research Institute's Growth Medium) [49] and incubated for seven days at 28 °C in a climatic growth chamber (28 °C, 12 h photoperiod, lightening of 150–200 µmol (photon) m^−2^ s^−1^) with moderate shaking. The supernatant was recovered by centrifugation at 4500 rpm for 15 minutes, and the solubilized phosphorus in the supernatant was measured using Olsen and Sommers colorimetric method [50].

#### 2.4.2. Indoles production

Indoles were estimated on liquid BG11 supplemented with tryptophan (0.2%). Cultures were incubated for seven days in a climatic growth chamber (28 °C, 12 h photoperiod, lightening of 150–200 µmol (photon) m^−2^ s^−1^). The cultures were then centrifuged, and 1 mL of the clear supernatant was combined with 4 mL of the Salkowski reagent [51]. After 30 minutes of incubation in the dark, the appearance of a pink color indicated a positive result for the production of indoles, and the measurement of the optical density was at 530 nm (SPEKOL 1300 UV VIS spectrophotometer, Analytik Jena, Jena, Germany). IAA (Sigma, St. Louis, MO, USA) was used as a standard (y = 0.0089x + 0.0113; R^2^ = 0.9975), and results were expressed as µg IAA mL^−1^ equivalents in average.

#### 2.4.3. Manganese solubilization

The screening of Mn-oxide-solubilizing ability was carried out in a manganese basal solid medium (MMB) containing MnO_2_ (0.5% and 1%) [52]. Plates were incubated in anaerobic jars using anaerobic generation kits (Oxoid, Basingstoke, UK) under microaerophilic conditions at 28 °C with illumination for 20 days required to form visible colonies in the plates. The MMB discoloration was used to determine the Mn oxide-solubilizing capacity.

#### 2.4.4. Hydrocyanic and ammonia production

To estimate hydrogen cyanide, each isolate was streaked on BG11 solid medium supplemented with glycine (4.4 g L^−1^). Each inoculated Petri dish was covered with a piece of Whatman paper of the same diameter soaked in picric acid (0.5%) and sodium carbonate (2%) solutions. The plates were covered with parafilm and incubated under optimum growth conditions. The change in color of the paper from yellow to orange or brown was used as an indicator of hydrogen cyanide (HCN) production [53].

Ammonia production was tested in peptone water inoculated with the different isolates. After seven days of incubation at 28 °C, 0.5 mL of Nessler reagent was added to each tube. The development of a yellow-to-orange color indicated a positive result [54].

#### 2.4.5. Siderophore production

The ability of the isolates to produce siderophore was assessed using the standard CAS test [55]. Glassware was rinsed with 3 M hydrochloric acid (HCl) to remove iron and then washed with deionized water [56]. The CAS reagent was prepared as follows: 121 mg of CAS was added to 100 mL of deionized water, and 20 mL of 1 mM ferric chloride (FeCl_3_6H_2_O) solution was prepared in 10 mM HCl. This solution was added by stirring to 20 mL of hexadecyltrimethylammonium bromide (HDTMA) solution prepared by dissolving 729 mg of HDTMA in 400 mL of distilled water. The modified microtiter plate method was used to determine siderophore production. The supernatant (100 µL) obtained from the culture broth of each isolate n was added to the different microtiter plate wells, followed by the addition of 100 µL of CAS reagent. After incubation, the optical density was measured at 630 nm using a spectrophotometer (SPEKOL 1300 UV VIS spectrophotometer, Analytik Jena, Jena, Germany). Siderophore was calculated as follows (Eq 1): Ar = Absorbance of the control, As = absorbance of the sample [57].

$$Siderophore\;production=\frac{\left(Ar-As\right)}{Ar}\times 100$$ (1)

### 2.5. 1-Aminocyclopropane-1-Carboxylate deaminase estimation

The ACC deaminase activity of the isolates tested was evaluated according to the procedure described by Brígido and his colleagues (2015) [58]. 200 µL of each liquid culture was added to 15 mL of liquid BG11 and incubated for seven days under light conditions. The cultures were centrifuged, and the pellets of the different isolates were washed with a minimal DF (Dworkin and Foster) salt medium without a nitrogen source [59]. Cell pellets were resuspended in 15 mL of DF salt minimum medium containing 3 mM of ACC, cultured for three days at 30 °C with shaking, and then centrifuged and washed with 5 mL of 0.1 M Tris-HCl (pH 7.6). The cell suspensions were transferred to a 1.5 mL microcentrifuge tube. The cell pellet was recovered by centrifugation and used for the enzymatic assay. The pellet from each isolate was resuspended in 400 µL of 0.1 M Tris-HCl (pH 8.0), 20 µL of toluene, and 50 µL of cell lysate from each isolate was divided into three microtubes, two with 5 µL of ACC (0.5 M) and one acted as a negative control. A second negative control was also prepared containing 5 µL of 0.5 M ACC and 50 µL of 0.1 M Tris-HCl (pH 8.0). After vortexing, the ACC was added to the cell suspensions, and all tubes were incubated at 30 °C for 30 minutes. 500 µL of 0.56 M HCl was then added. The cells were centrifuged, and the absorbance of the reaction mixtures was measured at 540 nm (SPEKOL 1300 UV VIS spectrophotometer, Analytik Jena, Jena, Germany). The standard was a solution of α-ketobutyrate (Sigma) in 0.1 M TRIS-HCl (pH 8.0). Using a calibration curve of α-ketobutyrate (5, 10, 15, 20, and 25 µmol mL^−1^), the ACC deaminase activity of the isolates was assessed and expressed as µmol α-ketobutyrate h^−1^ mg protein^−1^.

### 2.6. Soil bio consolidation activity

A combination of the most relevant isolates C2, C3, and M1 was prepared to investigate the ability of the isolates to form conglomerates. The isolates were grown separately on a liquid BG11 medium and combined equally to achieve a final density of 10^6^ UFC/mL [60]. 5 mL of the consortium was mixed with 25 g of sand (Betonella) containing 5 mL of CaCl_2_ (20 g/L) and placed in sterile molds (ø = 6.5 cm). A calcifying strain CCALA 192 was used as a positive control, and an autoclaved inoculum was used as a negative control. Incubation was carried out at 30 °C for 21 days [35]. The different samples were examined by Scanning Electron Microscopy (SEM) to visualize the presence of microbial traces and calcite production. The control and bio-consolidated soil block samples were collected and mounted on adhesive tape. The samples were analyzed without a sputter coater. Observations were made using a Gemini SEM 500 (Zeiss, Oberkochen, Germany). Sample images were visualized in BSE mode, 20 Pa pressure-vacuum, 15 Kv voltage, and a working distance of 9 mm modified according to optimal viewing conditions.

### 2.7. In planta bio stimulating capacity on Helianthus annuus

The consortium of the most relevant isolates was used for the *in planta* experience on *Helianthus annuus*. Sunflower is an excellent plant for studying biostimulating capability because of its high biomass production, expansive root system, and economic significance [61]. The seeds were surface sterilized with a sodium hypochlorite solution and rinsed several times with sterile distilled water [62],[63]. Inoculation was performed by immersing the sunflower seeds in the consortium (10^6^ CFU/mL) for 1 hour. The control plants consisted of seeds immersed in autoclaved inoculum. A salt stress of 5% (w/v) NaCl was applied to assess the isolates' ability to promote salinity stress tolerance in sunflower plants [64],[65]. Inoculated and non-inoculated seeds were sown in ordinary soil with a mixture of peat-based compost and vermiculite, and each experimental condition consisted of 30 pots for three replicates. Seedlings were thinned to one per pot after an emergency and grown in a greenhouse under a natural photoperiod. The maximum day and night temperatures were 30 and 20 °C, respectively. Thirty days after sowing (DAS), the plants were sampled, and shoots and roots were collected independently. The following parameters were analyzed: Germination percentage, shoot and root length, and chlorophyll content. The amounts of chlorophyll a, b, and total chlorophyll were measured using the method described by Arnon (1949) [66]. Briefly, 0.5 g of finely chopped areal parts from each sample were homogenized in 10 mL of acetone (80%) and stored overnight at −10 °C. After centrifugation, the absorbance of the supernatant was measured at 645 and 663 nm. The concentrations of chlorophyll a, b, and total in the samples were determined using the following formulate (Eqs 2–4 below):

$$Ch_{a}\;(mg\;L^{-1})=12.41\;(OD\;663)-2.59\;(OD\;645)$$ (2)

$$Ch_{b}\;(mg\;L^{-1})=22.9\;(OD\;645)-4.68\;(OD\;663)$$ (3)

$$Ch_{tot}\;(mg\;L^{-1})=Cha+Chb$$ (4)

### 2.8. Statistical analysis

Each experiment was performed in triplicate, and data are presented as mean ± standard deviation. Data were tested for normality using the Shapiro-Wilk test and analyzed using one-way analysis of variance (ANOVA) and Kruskal-Wallis as a non-parametric test, with significance differences determined at 5% probability. All analyses were performed using the XLSTAT 2016 program (Addinsoft, Paris, France). Primer 7 and PAST 4.03 software allowed the realization of taxonomic bar plots of ASVs at the phylum (1%) and genus (1%) level as well as the calculation of alpha diversity metrics (i.e., Simpson, Shannon, and Chao1 indices) for different samples.

## 3. Results

### 3.1. DNA extraction, 16S rRNA gene metabarcoding

The 16S rRNA gene metabarcoding results were used to investigate the diversity of the samples. As shown in Table 1, the sulfur pond samples contained 774 taxa numbers and 20963 individuals. The samples showed high diversity and species richness (Shannon H' index = 6 and Chao-1 = 774.1) with good homogeneity (Simpson 1-D close to 1). A moderate evenness value was obtained, indicating a relatively uniform diversification of the microbial community with respect to the main taxa.

**Table 1.** Diversity indices generated on 16S rRNA metabarcoding outputs using PAST 4.03.

Indice	value
Taxa S	774
Individuals	20963
Simpson 1-D	0.9947
Shannon H'	6.013
Evenness	0.5281
Chao-1	774.1

**Figure 1.**
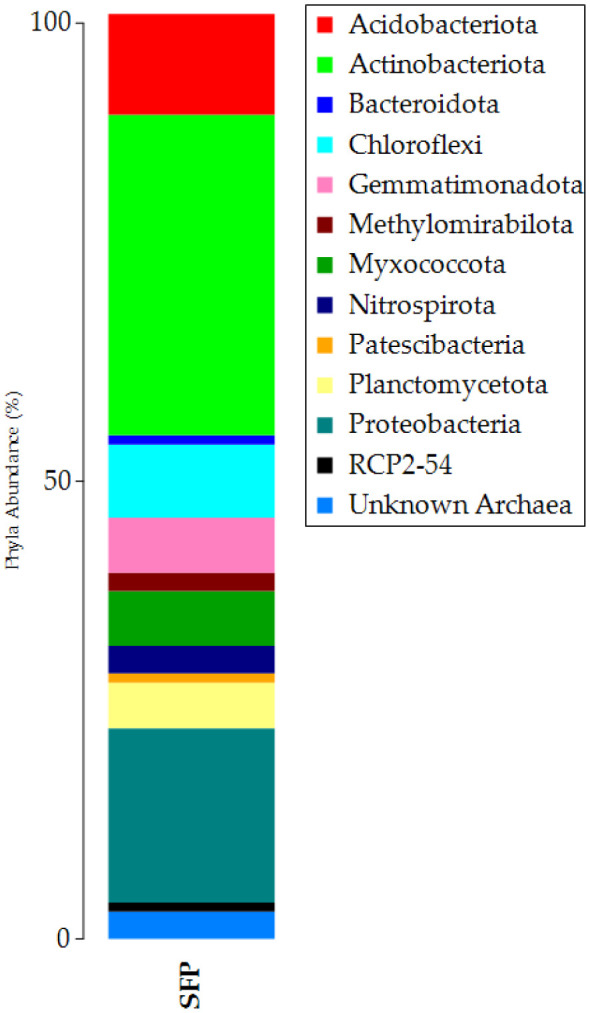
Taxonomic bar plot of the relative abundances of ASVs (Amplicon Sequence Variants) at the phylum level (1%).

To investigate the composition of ASVs at different taxonomic levels (phylum and genus), the results were filtered (1% cut-off) and presented with taxonomic bar plots. The abundances of ASVs at the phylum level are shown in Figure 1. The Bacteria domain was represented by 12 phyla, with Actinobacteria (syn Actinomycetota) being the most abundant phyla (relative abundance = 35%), followed by Proteobacteria (syn Pseudomonadota) (19%) and Acidobacteriota (11%). Other phyla were also notable: Chloroflexi (syn Chloroflexota), Gemmatimonadota, Myxococcota, and Planctomycetota (6% on average).

**Figure 2.**
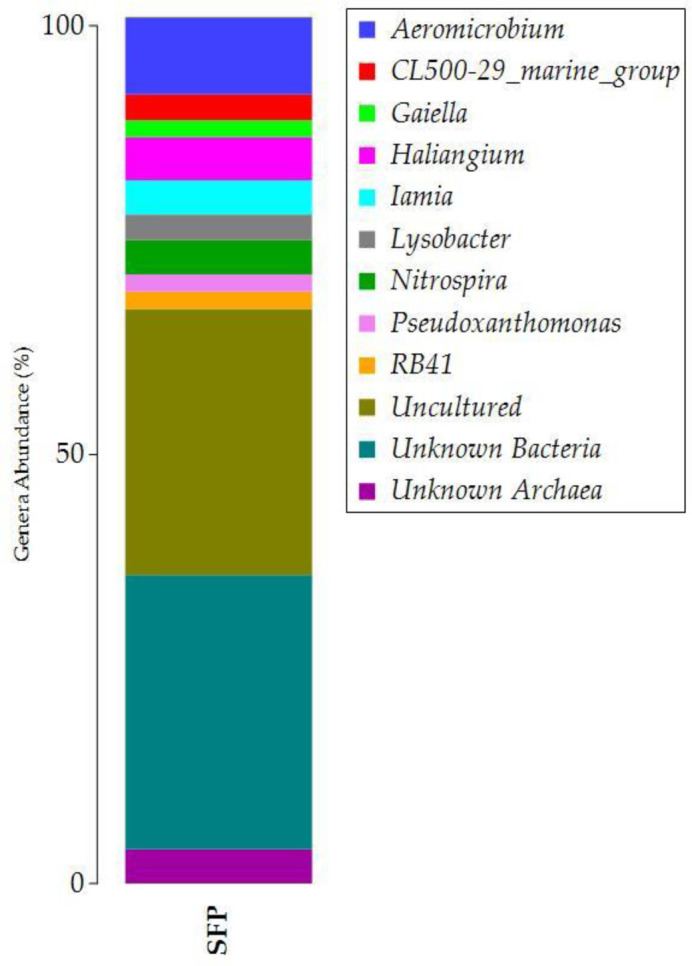
Taxonomic bar plot of the relative abundances of ASVs (Amplicon Sequence Variants) at the genus level (1%).

The microbial community at the genus level was filtered at 1% (Figure 2), which allowed us to keep the important genera in terms of percentage of abundance occupied from the total community (69% of significant genera and 31% of less abundant genera). The abundant ASVs were associated with *Unknown* and uncultured taxa (32 and 31% of relative abundance, respectively). The genus *Aeromicrobium* represented 9% of the important genera, followed by *Haliangium*, *Iamia*, and *Nitrospira* (4% on average). However, the *Unknown* Archaea represented 4% of the relative abundance of the total important community.

### 3.2. Isolation of culturable phototrophic microbiota

**Figure 3.**
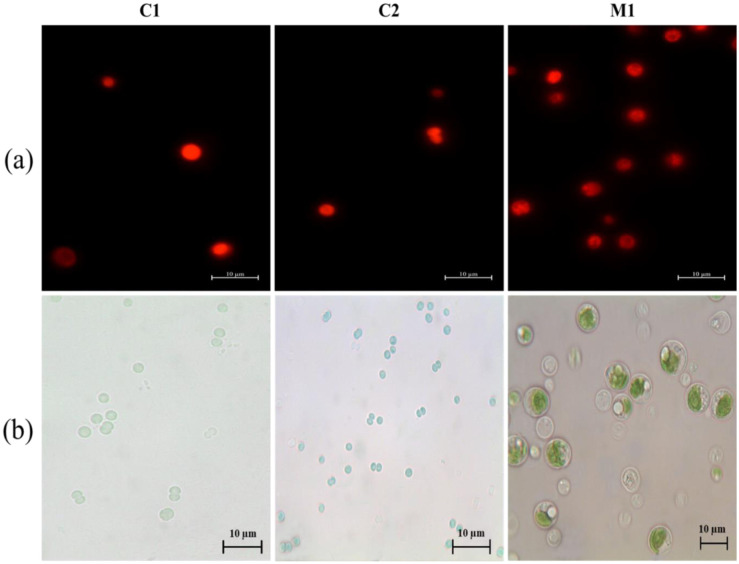
Fluorescence microscopy images of cyanobacterial and microalgae isolates overlay of optical microscopy images (a and b respectively) obtained at 100X. The cyanobacterial strains (C1 and C2) are homogenous colored under fluorescence microscopy beside the red autofluorescence of chlorophyll in the chloroplasts is evidence for the microalgae (M1). Scale bar 10 µm.

The isolation and purification of the isolates were carried out on BG11 agar medium. Eight isolates were obtained based on the cells' dimensions, morphology, and sensitivity to Lugol staining and fluorescent microscopy observations (C1, C2, C3, C4, M1, M2, M3, and M4). All the isolates showed mineralization ability. Microscopic observations of the isolates used for the consortium are reported below (Figure 3). Based on these results, the isolates were assigned to cyanobacteria (*Synechocystis* sp. C1, *Synechocystis* sp C2, *Synechocystis* sp C3, and *Synechocystis* sp C4) and microalgae (*Chlorella* sp. M1, *Chlorella* sp. M2, *Chlorella* sp. M3, and *Chlorella* sp. M4) genera.

### 3.3. Plant growth-promoting activities

#### 3.3.1. Phosphate solubilization

**Figure 4.**
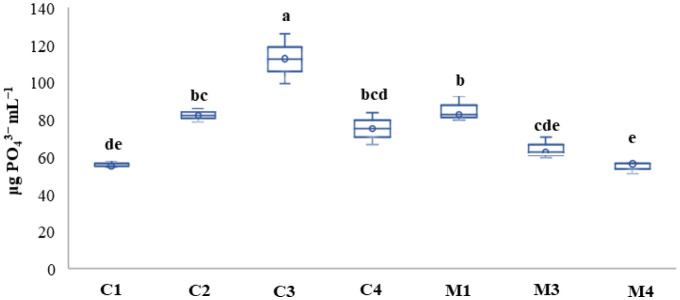
The amount of soluble phosphorus on liquid NBRIP obtained from the different isolates. Results followed by the same case letters are not significantly different according to Tukey's post-hoc test (p < 0.05) (n = 3). No result was obtained by M2 isolate.

The results for phosphate solubilization on liquid NBRIP are shown in Figure 4. Except for isolate M2, the majority of the isolates (87.5%) shared the capacity for phosphate solubilization with varying amounts depending on the isolate (p < 0.05). The highest solubilization was obtained by isolates C3, M1, and C2 (89.44, 85, and 82.26 µg PO_4_^3−^ mL^−1^ p < 0.05). The other isolates (C1, C4, M3, and M4) showed a significant solubilizing capacity (60.94 µg PO_4_^3−^ mL^−1^ p < 0.05) in mean, with the lowest value obtained by the M4 isolate (54.59 PO_4_^3−^ mL^−1^).

#### 3.3.2. Production of indoles

**Figure 5.**
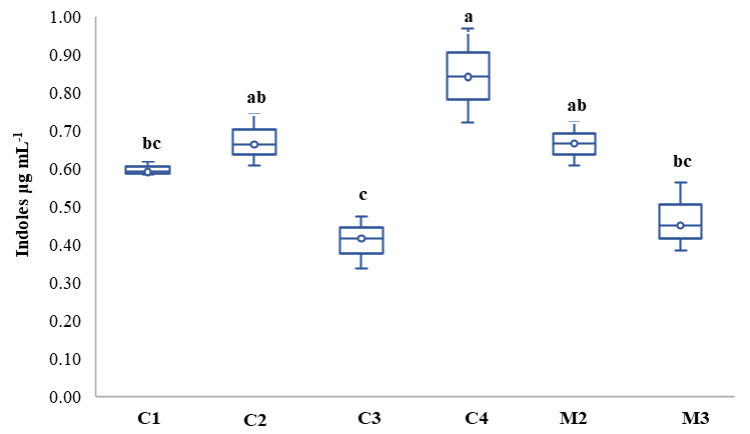
Indoles production by the different isolates. Results followed by the same case letters are not significantly different according to Tukey's post-hoc test (p < 0.05) (n = 3). No production was obtained by the M1 and M4 strains.

The quantitative estimation of indoles produced by the isolates is shown in Figure 5. The production was moderately expressed by six isolates, ranging from 0.41 to 0.84 µg mL^−1^ obtained by C3 and C4 isolates, respectively (p < 0.05). However, no production was signaled by M1 and M4 isolates.

#### 3.3.3. Manganese solubilization, Ammonia, Hydrocyanic acid, and siderophore production

The table below (Table 2) shows the different PGP activities expressed by the isolates: Manganese, ammonia, hydrogen cyanide, and siderophore production.

**Table 2.** Manganese tolerance, ammonia, hydrocyanic acid, and siderophore production by the different isolates.

Isolate/Test	Mn Tolerance 0.5%	Mn Tolerance 1%	NH_3_	HCN	Siderophore (%)
C1	−	−	++	+	0
C2	−	−	+++	+++	0
C3	−	−	++	++	0
C4	++	−	+++	−	0
M1	+++	−	++	−	0.23
M2	−	−	++	−	0
M3	−	−	+	−	4.75
M4	+	+	+	+	0

High activity (+++); medium activity (++); low activity (+); no activity (−).

As shown in the table, the isolates shared the ability to produce ammonia, with different intensities depending on the isolate. The HCN activity was expressed by C1, C2, C3, and M4 with moderate production. Regarding Mn tolerance, M1, C4, and M4 isolates grew in the presence of 0.5% of Mn in the medium. However, the M4 isolate tolerated up to 1% of Mn. Siderophore production was expressed by M1 and M3 isolates with 0.23 and 4.25% respectively.

### 3.4. ACC deaminase activity

The results for ACC deaminase activity are presented in the table below (Table 3). Almost all of the isolates (75%) have ACC deaminase activity, the highest amount being expressed by C1 and M1 isolates with 10.29 and 7.96 µmol α-KB mg proteins^−1^ h^−1^, respectively (p > 0.05). A moderate production was signaled by C3 isolate (4.00 µmol α-KB mg proteins^−1^ h^−1^). The remaining isolates C4, M3, and M4 showed the lowest value (2.14 µmol α-KB mg proteins^−1^ h^−1^) (P > 0.05).

**Table 3.** ACC deaminase activity expressed by the different isolates.

Isolate	ACC (µmol α-KB mg proteins^−1^ h^−1^)
C1	10.29 ± 0.65^g^
C3	4.00 ± 0.20^a^
C4	2.20 ± 0.14^f^
M1	7.96 ± 0.49^e^
M3	2.54 ± 0.19^c^
M4	1.70 ± 0.11^a^
K (observed value)	16.39
p-value (bilateral)	0.006

Results followed by the same lower-case letters are not significantly different according to Kruskal-Wallis test (p < 0.05) (n = 3).

### 3.5. Soil bioconsolidation

**Figure 6.**
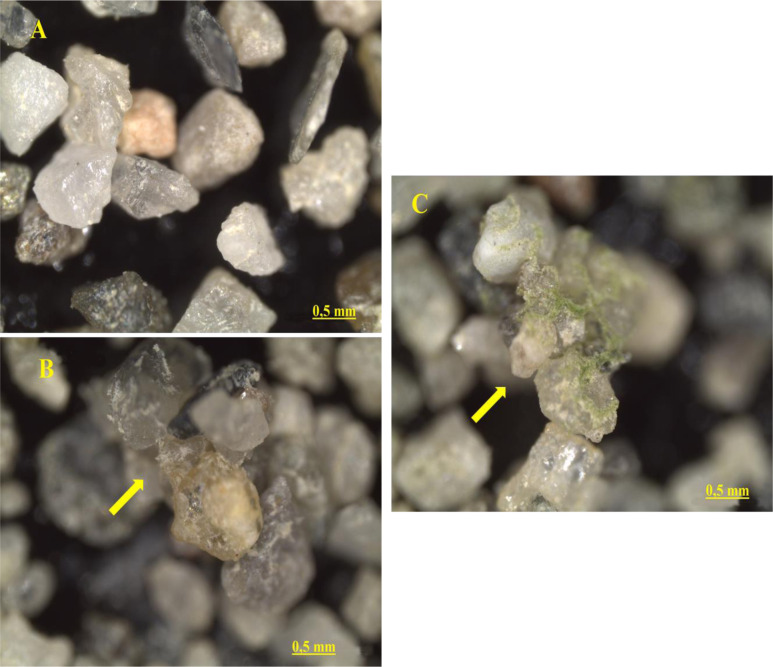
Stereomicroscopic images of sand particles at 4X magnification: (A) control sample, (B) sample treated with CCALA 192, and (C) consortium-treated sample.

The representative images of the different sand samples analyzed under the stereomicroscope are shown in the figure below (Figure 6). Other examples of bioconsolidation activity showing several consolidation points are presented in the supplementary material file (Figures S1 and S2). Both the consortium and the positive control (CCALA192) show the presence of biofilm, which binds sand particles together by forming bridges. In contrast, the control samples are separated and disconnected from each other.

**Figure 7.**
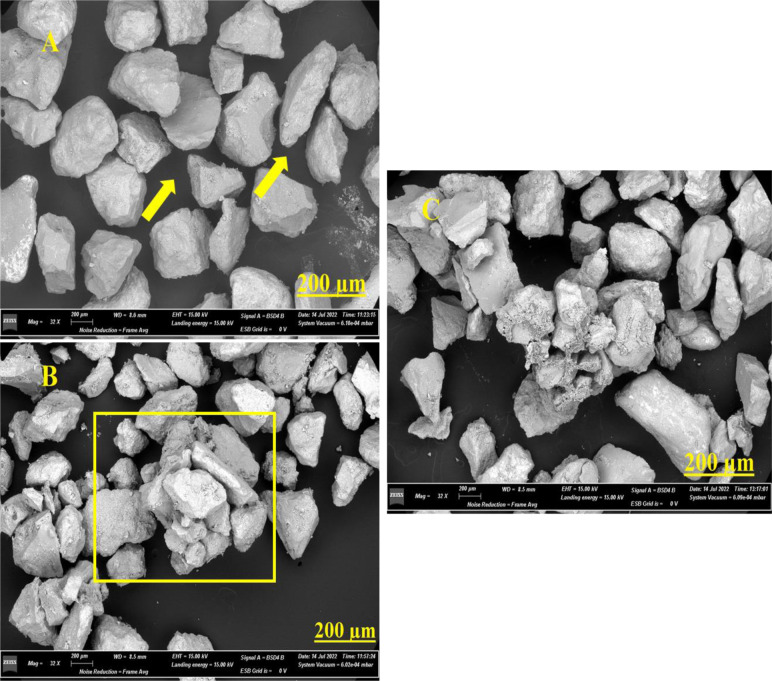
Scanning electron microscopy micrographs obtained at 32 X magnification: (A) control sample, (B) CCALA 192 sample, and (C) sample with consortium. The squares highlight the presence of conglomerated particles and biofilms.

Similarly, Scanning Electron Microscopy (SEM) visualization revealed an agglomeration of the sand particles in the treated samples and the positive control (CCALA192), whereas the control samples are disaggregated (Figure 7).

**Figure 8.**
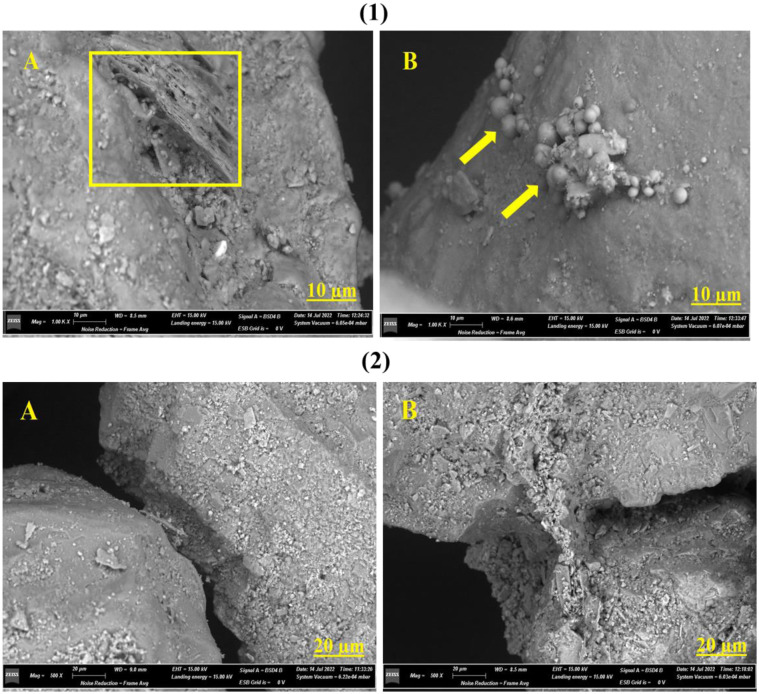
SEM micrographs obtained at 1.00 K X magnification (Figure 8.1); calcite sheets (1A) and cell agglomerates (1B). Figure 8.2 presents two detached siliceous matrices in the control sample at 500 X magnification (2A), and two siliceous matrices connected by calcium carbonate in the sample with consortium (2B).

The composition of these particle-related structures was further investigated by the SEM evaluation (Figure 8). The presence of specific leaflets indicates the presence of calcite within the sample particles treated with the consortium. For both the consortium and CCALA 192, vital structures of cellular agglomerates were found within the anchor bridges. These cells were spherical and small (1–2 µm).

Scanning Electron Microscopy investigation revealed positive *in vitro* results from the calcifying isolates, including the development of sand aggregates that were bridged together rather than the control samples that were separated and disconnected.

### 3.6. In planta bio stimulating capacity on Helianthus annuus

The plants' shoots and roots were collected separately at 30 DAS, and the following parameters were examined: Survival rate, shoot and root length, and chlorophyll content. The results are presented in the table below.

**Table 4.** Effect of the different treatments on plant biometric parameters and chlorophyll content.

	SR (%)	SL (cm)	RL (cm)	Chl tot	Chl a/b
CNT 0% NaCl	82.50^ab^	6.63^ab^	3.75^ab^	7.69^ab^	2.44^ab^
Cons 0% NaCl	88.50^b^	7.60^b^	9.35^b^	9.03^b^	2.77^b^
CNT 5% NaCl	33.00^a^	1.12^a^	0.75^a^	1.80^a^	0.50^a^
Cons 5% NaCl	84.25^ab^	4.00^ab^	2.15^ab^	2.30^ab^	1.91^ab^
K (observed value)	13.29	14.14	14.20	9.67	10.42
p-value (bilateral)	0.004	0.003	0.003	0.022	0.015

In the table, SR (Survival Rate), SL (Shoot Length), RL (Root Length), Chl tot (Chlorophyll tot), Chl a/b (Chlorophyll a/b). Results followed by the same lower-case letters are not significantly different according to Kruskal-Wallis test (p < 0.05) (n = 3).

During the greenhouse experiment, the inoculation with the consortium improved the growth of the plants under normal conditions and their tolerance to salt stress compared to the untreated plants (uninoculated control). Inoculated plants had better survival rates under normal conditions (82.5%) and with 5% salt stress (84.25%) compared to the control ones (p < 0.05).

Concerning shoot and root length. Without salt stress, the highest length was obtained by the plants treated with the consortium, followed by the control one (p > 0.05). Under salt stress, the inoculation has improved the aerial and radical lengths compared to the uninoculated plants (p > 0.05). The same trends were reported for chlorophyll content; the inoculated plants had higher total chlorophyll content than the control plants under normal conditions and salt stress (p > 0.05).

Without salt stress, no statistical difference was found for the ratio Chla/Chlb in both treated and control plants (p > 0.05). With the application of salt stress, this ratio increased in the inoculated plants (1.91) compared to the uninoculated ones (0.5), but they were not statistically different (p > 0.05).

## 4. Discussion

In recent years, next-generation sequencing technology has enabled a variety of approaches to study microbiomes based on the 16S gene. The phylum Actinobacteriota is one of the largest taxonomic units in terms of number and diversity among the major lineages currently recognized within the domain of Bacteria [67]. Actinobacteriota was the most abundant phylum in our survey (35% of relative abundance). The abundance of Actinobacteriota is determined by their ability to metabolize a wide range of carbon sources, from fresh substrates such as cellulose to highly complex ones such as polycyclic aromatics [68],[69]. The second most abundant phylum in the sample was Proteobacteria. This phylum is a typical representative in various ecosystems and plays a vital role in transforming organic compounds. The Proteobacteria phylum contains nitrifying and denitrifying genera, which are essential for recycling nutrients and remineralizing organic matter and are considered the most metabolically versatile [70]–[72]. Proteobacteria are generally facultative anaerobes, heterotrophic, low pH tolerant, and can be found in various environments. Many members of the genus can stimulate plant growth and development and increase plant resistance to biotic and abiotic stresses through nitrogen fixation, nutrient availability, and the production of phytohormones, antibiotics, and other extracellular metabolites [73]. The abundance of Acidobacteriota has been reported to reach up to 52% on some surfaces and an average of 20% in different soil environments [69]. In our study, it accounts for 11% of the relative abundance. Members of this phylum are tiny rod-shaped bacteria with the G morphotype. Based on DNA sequences, they are ubiquitous [74]. The Chloroflexi are mainly found in anaerobic environments, where they are crucial for the degradation of complex polymeric organic materials that promote the expansion of bacterial populations [73]. Members of the bacterial phylum Gemmatimonadetes are widespread in natural ecosystems, making it one of the nine most abundant phyla in soils [75]. Gemmatimonadetes are highly adapted to both oligotrophic and arid environments, according to diversity assessments based on 16S rRNA genes. Despite their widespread distribution, the physiology, ecology, and role of Gemmatimonadetes in environmental processes need to be better understood [76],[77]. Myxococcota are a remarkable bacterial phylum due to their distinctive social habits (e.g., predation and fruiting mass development), unique among prokaryotes [78]. Because of their versatility, Myxococcota are considered keystone taxa that play essential roles in microbial interaction pathways [79]. The phylum Planctomycetota includes environmentally and biotechnologically essential bacteria. Together with two other sister phyla (Planctomycetota-Verrucomicrobiota-Chlamydiota), it forms the superphylum PVC [80].

At the genus level, Unknown Bacteria and Uncultured were the most dominant in our study. Unknown microorganisms, sometimes called “microbial dark matter”, are statistically dominant in all major ecosystems on Earth. In addition, it is estimated that around 25% of the Earth's microbial population consists of taxa with no cultured ancestors. This means that ecosystem functioning could benefit from studying these previously unexplored organisms [81],[82]. Direct sequencing of environmental DNA revealed that most microbial lineages are uncultivated, severely limiting the ability to characterize their taxonomy and understand their biology [83],[84]. Considering all full-length 16S rRNA genes found in public databases, uncultured microbes-including those with high divergence- are quite common. Metagenomes had fewer 16S rRNA gene sequences from cultured species. The remaining 16S rRNA gene sequences were from uncultured genera and higher taxonomic groups [81]. Uncultured taxa are prevalent in the Earth's microbiome and often exhibit incredibly high levels of phylogenetic novelty. They may also harbor as yet unidentified ecosystem-level activities [81],[82]. *Aeromicrobium* was first described in 1991 [85]. It refers to a group of Gram-positive, aerobic, motile, rod-shaped bacteria. The genus belongs to the family Nocardioidaceae within the order Actinomycetales [86]. *Aeromicrobium* comprises 22 species, most isolated from different environments (air, soil, and water) [87]. *Haliangium* is a genus of bacteria belonging to Kofleriaceae family, whose members generate the antifungal compound haliangicins [88]. The first known halophilic myxobacterial taxa belong to the genus *Haliangium* [89]. Within the suborder Nannocystineae, Haliangiaceae is a distinct taxon of myxobacteria, while Kofleriaceae appears to be their closest related family. They inhabit a new and distinct phylogenetic cluster [90]. Myxobacteria have two distinguishing features. The first is their high capacity to produce secondary metabolites, most of which act on eukaryotic or prokaryotic cells and are therefore being explored for use in crop protection or medicine [91]. Their second characteristic is morphogenesis, which relies on cell-to-cell communication between individual cells in a swarm to form fruiting bodies and produce myxospores [92]. *Iamia* is one of the genera that are primarily beneficial and can stimulate plant growth by improving nutrient cycling [93]. However, the genus *Iamia* has yet to be thoroughly investigated for its importance in agricultural productivity [94]. *Nitrospira* is one of the major players in nitrite oxidation in freshwater environments [95]. Bacteria of the genus *Nitrospira* have also been found to occur in different habitats: Immunological methods have been used to identify *Nitrospira* in a variety of soil types, and a large number of similar bacteria have been recovered from freshwater aquariums, groundwater contaminated by animal waste, and nitrifying bioreactors and biofilms [96],[97]. Overall, the genera *Nitrobacter* and *Nitrospira* are widespread and could significantly impact global nitrite oxidation [98].

The *in vitro* results showed various plant growth-promoting properties of the isolates: phosphate solubilization, indoles, hydrocyanic acid, ammonia, siderophore production, and manganese solubilization. Auxins regulate photosynthesis, pigment synthesis, the production of various metabolites, and plant resistance to various environmental stresses. They also regulate vegetative development, flowering, and fruiting in plants. Among auxins, indole-3-acetic acid (IAA) is the most studied plant growth regulator regarding physiological, biochemical, and genetic properties [62],[65]. There is evidence that cyanobacteria and microalgae can release indoles, amino acids, and other substances into the environment that can reach the root cortex and promote the growth of soil microbial communities [99]–[101]. Some cyanobacteria and microalgae can secrete hydrogen cyanide, an antibacterial and antifungal compound that enables the biocontrol of phytopathogens [102]–[104]. It should be noted that the amount and quality of the substances excreted vary depending on the type of strain, their growth stage, and the environmental factors [105]. It is well known that many extracellular compounds, including plant growth regulators, vitamins, amino acids, carbohydrates, and other metabolites released by cyanobacteria and microalgae directly or indirectly affect plant growth and yield [105],[106]. In addition to their well-known roles as nitrogen suppliers and desiccation resistors, cyanobacteria and microalgae play an essential role in carbon sequestration, increasing agricultural yields and fertilizer use efficiency [31],[107],[108]. Ethylene is a gaseous growth regulator component important in many aspects of plant development, including their susceptibility to stress [109]. One mechanism used by many PGPMs to promote plant growth and development is the action of the enzyme 1-aminocyclopropane-1-carboxylate (ACC) deaminase to reduce plant ethylene levels [110]. Many bacterial and fungal species possess 1-aminocyclopropane-1-carboxylate deaminase (ACCD), a pyridoxal phosphate-dependent enzyme. ACCD converts ACC, a direct precursor of ethylene, to ammonia and α-ketobutyrate, thereby reducing levels of stress ethylene, which inhibits plant growth [111],[112]. Microorganisms containing ACC deaminase can help mitigate the damaging effects of stress-induced ethylene on plants [113]. In this work, most of the isolates (75%) have ACC deaminase activity. ACC deaminase has been extensively documented in many microbiological species, including fungi, rhizobia, endophytes, and Gram-positive and harmful bacteria [114]. Inoculation with PGPM having ACC deaminase activity can help maintain plant growth and development under stress conditions by reducing stress-induced ethylene synthesis [65]. Much scientific evidence shows that the ACC deaminase activity of ST-PGPM (salt tolerant PGPM) helps to improve plant survival in saline soils and increases plant productivity [111],[115].

The consortium formed by the most pertinent isolates efficiently consolidated sand particles in the presence of calcium carbonate by forming biomineralized aggregates. Bioconsolidation is used in geotechnical engineering to reduce or stabilize erosion and improve slope stability. Traditional methods of improving soil structure involve cement or chemicals. However, they can permanently contaminate the soil, water, or the air [116]. The chemicals can be injected into the ground to bind sand grains together, making the soil stiffer and more durable. However, this approach is expensive, difficult to apply evenly, and introduces harmful substances into the soil [37]. It is vital to find a practical approach to improving soil quality. The term “Microbially Induced Calcite Precipitation” (MICP) describes the process by which microbial cells and metabolic activity cause calcium carbonate precipitation from a supersaturated liquid [116]. However, the most common technique for calcium carbonate precipitation involves urea hydrolysis [37],[116]. MICP has been researched as a desirable grouting technique to improve soil structure and the durability of building and cementing materials [116]. Sand grains are bound together by particle-particle interactions when CaCO_3_ precipitation is induced, making the soil stiffer and stronger [117]. Bioconsolidation can lead to a tenfold increase in the sand's porosity, stiffness, compressibility, and shear strength [37]. The stereomicroscope and SEM images showed the formation of bridges linking the sand particles together. The CO_3_^−2^ ions were found to precipitate with Ca^2+^ as calcite crystals, forming cementing bridges between the sand particles [116]. Microorganisms may have acted as nucleation sites during the mineralization process, as evidenced by the calcite crystals formed between soil particles and by the presence of embedded microbes [118]. Several studies have shown that inoculation with *Nostoc* sp., *Scytonema* sp., *Microcoleus vaginatus*, *Phormidium* sp., and microalgae increases soil aggregation on sand [119]–[121].

The isolates used in this study exhibited various plant growth-promoting activities *in vitro*. The greenhouse experiment positively affected plants inoculated with the consortium under normal and salt stress conditions. Considering their compatibility, formulating a multi-strain bacterial consortium facilitates synergistic communication among the bacterial strains, reducing inhibitory by-products and enhancing balanced plant nutrition, thereby promoting plant growth and development in diverse environments [122],[123]. A multi-strain consortium enhances plant development more effectively than a single strain due to the synergistic effects of diverse mechanisms employed by multiple microbial strains [124].

Cyanobacteria and microalgae respond to salinity stress by promoting plant growth and development. A multitude of mechanisms is involved, including Nitrogen fixation, accumulation of compatible solutes, synthesis of plant growth hormones, activity of 1-aminocyclopropane-1-carboxylate (ACC) deaminase, generation of exopolysaccharides, synthesis of siderophores, production of hydrogen cyanide, and defense enzymes [125]–[127]. The release of phytohormones, such as auxins and gibberellins, by cyanobacteria and microalgae has been shown to benefit plant growth and development [101],[128],[129]. Cyanobacteria and microalgae produce a variety of metabolites that can help regulate plant growth under abiotic stress conditions such as salinity [99],[130]. According to Katoh and his collaborators (2012) [131], germination-promoting substances produced by cyanobacteria are responsible for several vegetable plants' increased development and rooting. Similarly, Gharib and his collaborators (2024) show the efficiency of foliar spraying of various microalgae on common bean plants' growth and development [101]. Cyanobacteria and microalgae have been shown to release various physiologically active chemicals extracellularly, which function as signalling molecules to support plant growth and improve soil nutrient levels [101],[130],[132],[133]. There are reports of cyanobacteria and microalgae being used to restore salt-damaged soils [99],[134],[135]. Cyanobacterial and microalgae inoculation have improved abiotic stress tolerance in various crops [99],[136].

## 5. Conclusions

Cyanobacteria and microalgae are attracting increasing interest from the scientific community and the agrochemical industry as novel renewable sources of plant biostimulants that can consistently enhance the quality and yields of agricultural supplies. This work demonstrated the effective screening of cyanobacterial and microalgal strains with different plant growth-promoting properties and ACC deaminase activity. The consortium of the most relevant isolates showed good *in vitro* bioconsolidation ability of sand particles in the presence of calcium carbonate, forming biomineralized aggregates. The ability of the consortium to induce halotolerance in sunflower plants was tested in a greenhouse experiment. Under normal and stressful conditions, the consortium positively affected sunflower germination and growth. These results emphasize the need to carefully select a suitable consortium based on primary *in vitro* tests. Furthermore, the ability to construct biocrusts highlights the importance of the research in providing a comprehensive, flexible, and efficient framework for decision-making in the restoration of drylands. This promotes long-term soil fertility and stability, lowering the demand for artificial fertilizers and mitigating environmental deterioration. The study results suggest that a cyanobacteria-microalgae consortium could be a viable option for enhancing biocrust formation, plant development, and salt stress tolerance, providing a long-term and efficient solution for promoting plant growth in challenging environments. Our findings demonstrate the potential for using cyanobacteria and microalgae in sustainable agriculture. Their beneficial effects on plants and soil demonstrate their efficacy as bioconsolidant and biostimulant agents, providing a viable approach to environmental preservation and sustainable agriculture. Further research is needed to investigate the efficacy of cyanobacteria and microalgae inoculation in open-field experiments with different plant species and under challenging conditions. In addition, a practical decision-making system based on biocrust for the regeneration of arid environments is needed. The results provide a valid scientific basis for demonstrating the possibility of using these strains as bioconsolidation agents. The evidence is also useful for other applications where bioconsolidation is of interest. For example, the results of this research may pave the way for further work on the use of cyanobacteria and microalgae in sustainable agriculture, bioremediation, bioconstruction, and heritage preservation.

## Use of AI tools declaration

The authors declare they have not used Artificial Intelligence (AI) tools in the creation of this article.


