# Supplementary material for: Isolation and characterization of cyanobacteria and microalgae from a sulfuric pond: Plant growth-promoting and soil bioconsolidation activities
Source: AIMS Microbiol. 2024 Nov 8;10(4):944–72. doi: 10.3934/microbiol.2024041 (PMC11609429; doi:10.3934/microbiol.2024041)
Supplement: Supplementary file 1 [file microbiol-10-04-041-s001.pdf]

Microbiology

AIMS Microbiology, 10(4): 944–972.
DOI: 10.3934/microbiol.2024041
Received: 17 May 2024
Revised: 24 September 2024
Accepted: 21 October 2024
Published: 08 November 2024

https://www.aimspress.com/journal/microbiology

Research article

Isolation and characterization of cyanobacteria and microalgae from a sulfuric pond: Plant growth-promoting and soil bioconsolidation activities

Beatrice Farda[1], Rihab Djebaili[1,*], Enrico Sabbi[1], Giancarlo Pagnani[2], Paola Cacchio[1] and Marika Pellegrini[1]

1 Department of Life, Health and Environmental Sciences, University of L'Aquila, 67100 L'Aquila, Italy
2 Department of Bioscience and Technologies for Food, Agriculture and Environment, University of Teramo, Teramo, 64100, Italy

* **Correspondence:** Email: rihab.djebaili@univaq.it; Tel: +390862433246.

Supplementary

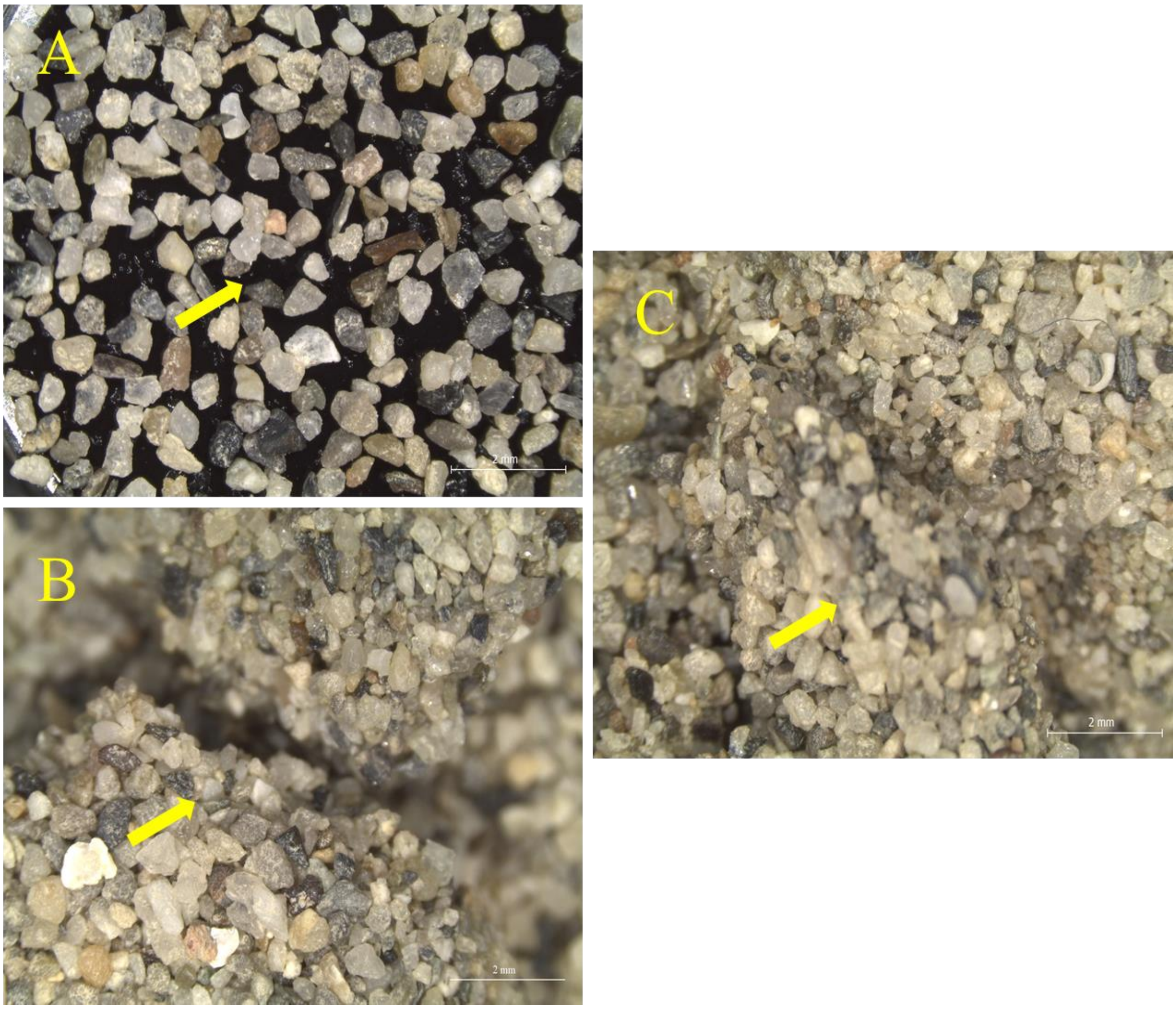

Figure S1. Stereomicroscopic images of sand particles at 1X magnification: (A) control sample, (B) sample treated with CCALA 192. and (C) consortium-treated sample.

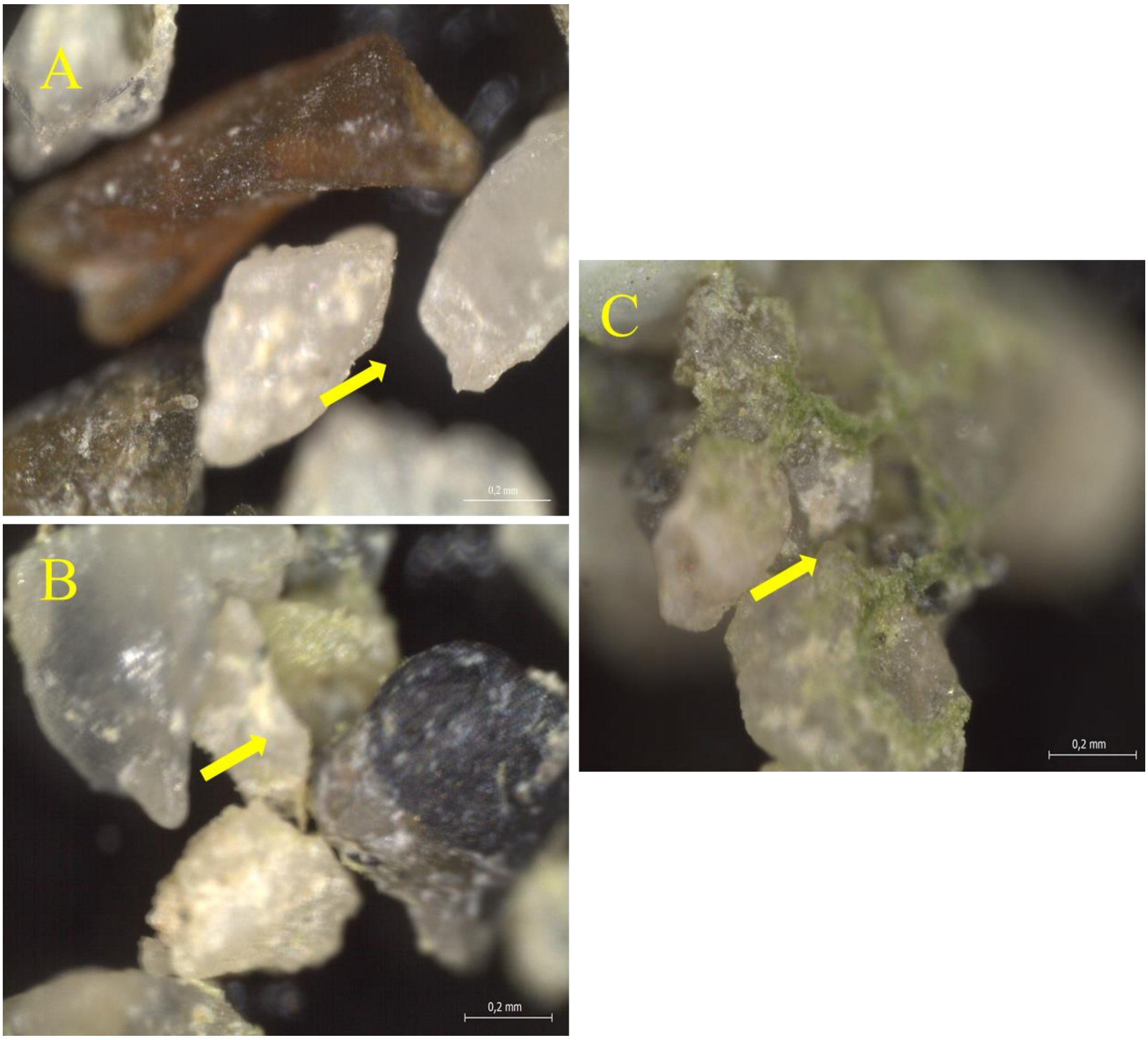

Figure S2. Stereomicroscopic images of sand particles at 8X magnification: (A) control sample, (B) sample treated with CCALA 192. and (C) consortium-treated sample.

AIMS Press

© 2024 the Author(s), licensee AIMS Press. This is an open access article distributed under the terms of the Creative Commons Attribution License (https://creativecommons.org/licenses/by/4.0)
